# Analyses of historical documents reveal past trends of exploitation of manatees ( *Trichechus inunguis*) in the Amazon Basin (16th-19th centuries)

**DOI:** 10.12688/openreseurope.20811.1

**Published:** 2025-08-18

**Authors:** Cristina Brito, Ana Catarina Garcia, John Nicholls, Jaime Silva, Nina Vieira

**Affiliations:** 1 CHAM and Departamento de História, Faculdade de Ciências Sociais e Humanas, FCSH, Universidade NOVA de Lisboa, Lisboa, 1069-061, Portugal; 2 CHAM, Faculdade de Ciências Sociais e Humanas, FCSH, Universidade NOVA de Lisboa, 1069-061 Lisboa, Universidade NOVA de Lisboa, Lisboa, 1069-061, Portugal; 3 Trinity Centre for Environmental Humanities, A6.003, Trinity College Dublin, 2 College Green, Dublin 2, D02 PN40, Ireland

**Keywords:** Accelerated extractions; Aquatic megafauna; Brazil; Colonisation and extirpations; Marine environmental history; Marine exploitation and use of resources.

## Abstract

The West Indian manatee ( *Trichechus manatus*) and the Amazonian manatee ( *Trichechus inunguis)* have been exploited by different societies from ancient times to the present. Deploying the matrix of Indigenous knowledge and uses, the hunting of manatees was at the core of European colonisation strategies of the Americas within a framework of appropriation, use, and consumption of aquatic mammals spanning the (post)colonial period. Today, both species are listed by the International Union for the Conservation of Nature and Natural Resources (IUCN) as Vulnerable, and population trends are expected to decrease in the future. While the exploitation of manatees (in particular, *T. inungis*) is well described in the 20th century, no overall quantitative assessments of such exploitation in the preceding centuries have been made. Here, we address this gap of knowledge through a systematic review of the literature, identification and review of documentary sources, selection and extraction of quantitative data, and contextual analysis through cross-analysis of historical documents of several types. Our quantitative estimates resulted in a total of 14.030 individuals captured, which are most probably largely underestimated. It corresponds to a biomass removal of 6.248,9 tonnes in a period of non-continuous 52 years between the 16 ^th^ and the 19 ^th^ centuries. Specifically, an average of c. 14.000 manatees were hunted between 1843 and 1898, comprising 91% of all captures prior to the twentieth century when another point of accelerated extraction occurred. We determined a trend of increasing captures from the 17 ^th^ to the 20 ^th^ centuries, for Brazil, but an important lacuna of data for the 16 ^th^ century, and pre-European contact, persists. However, the results inform current and future conservation measures for the species, while also including historical, cultural, traditional, and indigenous perspectives about the use of aquatic resources.

## Abstract (es)

El manatí antillano ( *Trichechus manatus*) y el manatí amazónico ( *Trichechus inunguis*) han sido explotados por distintas sociedadesThe West Indian manatee ( *Trichechus manatus*) and the Amazonian manatee ( *Trichechus inunguis)* have been exploited by different societies from ancient times to the present. Deploying the matrix of Indigenous knowledge and uses, the hunting of manatees was at the core of European colonisation strategies of the Americas within a framework of appropriation, use, and consumption of aquatic mammals spanning the (post)colonial period. Today, both species are listed by the International Union for the Conservation of Nature and Natural Resources (IUCN) as Vulnerable, and population trends are expected to decrease in the future. While the exploitation of manatees (in particular, *T. inungis*) is well described in the 20th century, no overall quantitative assessments of such exploitation in the preceding centuries have been made. Here, we address this gap of knowledge through a systematic review of the literature, identification and review of documentary sources, selection and extraction of quantitative data, and contextual analysis through cross-analysis of historical documents of several types. Our quantitative estimates resulted in a total of 14.030 individuals captured, which are most probably largely underestimated. It corresponds to a biomass removal of 6.248,9 tonnes in a period of non-continuous 52 years between the 16 ^th^ and the 19 ^th^ centuries. Specifically, an average of c. 14.000 manatees were hunted between 1843 and 1898, comprising 91% of all captures prior to the twentieth century when another point of accelerated extraction occurred. We determined a trend of increasing captures from the 17 ^th^ to the 20 ^th^ centuries, for Brazil, but an important lacuna of data for the 16 ^th^ century, and pre-European contact, persists. However, the results inform current and future conservation measures for the species, while also including historical, cultural, traditional, and indigenous perspectives about the use of aquatic resources.

## Introduction

*There is a certain fish, which we call marine ox, the Indians call it iguaraguá, frequent in the Captaincy of Espírito Santo [Brazil] (…); it feeds on herbs as indicated by the chewed grasses caught in the rocks bathed by mangroves. Exceeds the ox in corpulence; it is covered with hard skin, resembling the color of an elephant; it has two arms close to the breasts with which nothing; and under them teats with which their own children suck; it has a mouth entirely similar to that of an ox. It is excellent for eating, you would not know how to discern whether it should be considered as meat or rather as fish; from its fat, which is inherent in the skin and especially around the tail taken to the fire, a sauce is made, which can be compared to butter and I do not know if it will exceed it; its oil is used to spice up all foods: your whole body is full of solid and very hard bones, such that they can act as ivory* ^1^. ^1^

There is an established history of the hunting of manatees ( *Trichechus* spp.), or sea cows, which are known as ‘peixe-boi’ or ‘peixe-mulher’ in Portuguese, as ‘manati’ in Spanish, or as 'goaragoa' in old Tupi ^2, 3^. It has occurred in geographic ranges of different species’ historical presence and in different human societies that live with and exploit them.

**Figure 1.**
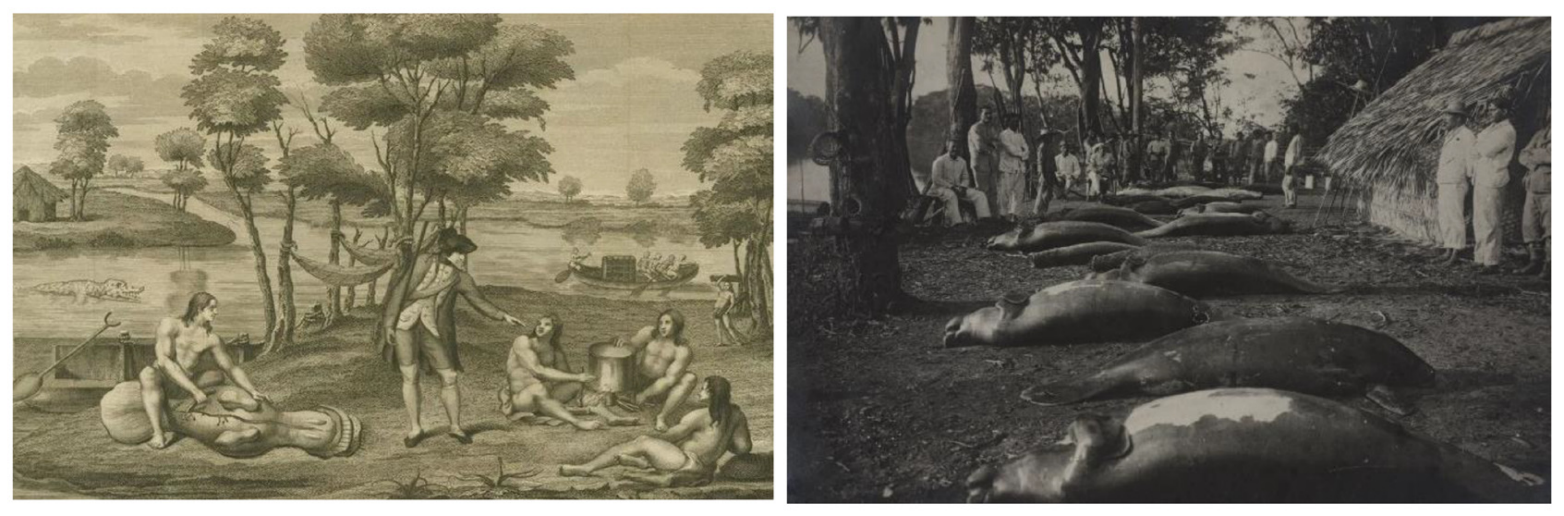
Left: A scene of a colonial setting in a Central or South American region (18th century) showing the butchering of a manatee and possibly the boiling of its fat. Iconographic sources add information to documentary sources and indicate the relevance of an animal and aquatic resource for a given historical and cultural context. Open Access © The John Carter Brown Library at Brown University (Permalink: https://jcb.lunaimaging.com/luna/servlet/s/4io7aw). **Right** : Photography of manatee hunting in Lake Ayapua, the Purus River (Amazonia) of unknown date. Images and photographs are visual sources that indicate the relevance of aquatic and animal resources for a given historical and cultural context. Permission obtained from Samuel Iglesias from https://www.delcampe.net/fr/collections/.

Manatee historical capture and use are reported in several regions of South America and the Caribbean basin where exploitation occurred long before the post-Columbian era ^4, 5^. In the Amazon basin, with its tributaries and adjacent marine region, manatees have been important resources for the subsistence of indigenous peoples and colonial settlements. At first, they were used for food, raw material for objects, and cultural elements in an interconnected way of living in the world ^2, 6^. Historically, they have been local food, staples, and medicinal products. Long-term capture, consumption, and use of manatees is evident from animal bones found in shell mounds, an ongoing research project on Amazonian *sambaquis* that has revealed the presence of manatee bones, and artefacts, since at least the middle and late Holocene ^7– 10^. Before European colonisation, manatee habitats were densely populated by many different indigenous societies, and the two sides of the Amazon were occupied, transformed by agriculture and the waters exploited both locally and regionally. The population density described by European chroniclers, such as the Jesuit father Alonso de Rojas, Diogo Nunes or Cristóbal de Acuña, about the first contacts indicates an advanced ability of the indigenous peoples to catch, produce and store food ^11, 12^. Among the storage techniques were small maize granaries, manioc flour and salt, the latter for salting fish, which indicated a certain degree of preservation capacity. The presence of turtle nurseries, many hunting and fishing methods, and well-known fishing grounds was abundantly described upon the arrival of Europeans in the American territories ^11– 14^. In particular, the entire area between the Orinoco and Amazon Rivers, which the Spaniards called Isla Guayana, was a vast landscape dominated by the Amazon tributaries, a vast dense forest, and various fauna. In the case of manatees, they were captured and used by pre-European contact Indigenous societies and continued to be exploited throughout the centuries to come ( Figure 1).

For the conquest and colonisation process, access to natural resources was essential for European survival ^15^. The European conquest of the Amazon from the 15 ^th^ century, first along the shores of the river mouth by the Portuguese, rapidly moved into the hinterland following the complex watercourses of the Amazon and its multitude of tributaries ^14, 16^. This process was marked by the construction of control posts and forts during the colonial period. At the beginning of the 17 ^th^ century, several European powers fought for control of the Amazon. Some of the first fortifications along the Amazon were built by the Dutch and the English, who, like the Portuguese, tried to control the region.

Due to the difficulty of transporting and maintaining European domestic animals in the Amazon hinterland, the first settlers had to look for local alternatives and negotiate with local communities to obtain access to resources ^17, 18^. The consumption of certain aquatic species by Europeans, such as manatees, turtles, and several river fish, eaten dry or salted, solved the problem of animal protein needs, which in the European context was supplied by cattle. That meant, along with other supplies, access to the river's resources such as fish, turtles, and marine mammals. In this context, contact with and observation of the practices of indigenous peoples, their daily uses of resources, and consumption were crucial to the survival of the first European settlements in the region. To gain access to the amounts needed to support newly established colonial societies, local fishing communities were subjugated through a process of appropriation of traditional knowledge and techniques and labour for hunting and fishing ^19, 20^. As the centuries passed, manatees became symbolic elements for the establishment of human interactions with the natural world from a positive perspective; manatees are established in local myths and legends and are part of Amazonian and Brazilian folklore. But there was also a negative context; manatees are now designated Vulnerable to extinction in the near future due to both the intensive exploitation in the recent past, as well as habitat degradation and human intrusion from continuing industrial development in the present. ^2^ Manatees, among countless other aquatic megafauna species, typify the recurrent historical narrative of exploitation and unsettling of the local nature ^2^.

It is increasingly recognised that imperialism and colonization have transformed natural environments, on differing scales and intensities at a global level, and there are still significant gaps in knowledge about the patterns, causes, and consequences of these processes of appropriation, transformation, and exploitation ^21^. This is particularly evident in the case of the Portuguese imperial context, which remains little studied and rarely included in specialised works. Compared to other imperial nations, such as the British Empire, which has been generously documented by academics ^22^, this article seeks to populate the lacuna of Portuguese Imperial and Colonial knowledge. Our primary goals are to use historical and geographical information to establish a trajectory of past exploitation of manatees ( *Trichechus* spp *.*) in South America for the 16 ^th^, 17 ^th^, 18 ^th^, and 19 ^th^ centuries, and to address narratives of change in the exploitation and use of aquatic resources. This article also aims to identify and quantify manatee extractions, mainly for the Amazonian manatee ( *Trichechus inunguis*), historically based on review of the scientific literature, reports, logbooks, treatises, and other documents.

## Methods

We conducted a four-step approach that included a literature review, identification, and review of documentary sources, the mining of existing data (i.e. selection and extraction of quantitative data, and identification of geographic location cartographic sources). All historical sources and data collected and used in this study to estimate manatee extraction in the Amazon basin are available in online, open-access format that summarises the data sets and provides statistical information in the Supplementary Material ^23^ (see also Table 1).

**Table 1.** Number of extracted individual manatees and biomass. See, for detailed data and sources: Vieira, N., Brito, C., & Nicholls, J. (2024). Extraction Amazonian Manatee ( *Trichechus inunguis*), 1533-1898 [Data set]. Zenodo. https://doi.org/10.5281/zenodo.15739198.

Year	Ave_individualCount	MAX Number of manatees	MIN Number of manatees	organismQuantity [biomass in ton]
1533	2	2,0	2,0	1,1
1606	3	3,9	1,7	1,5
1608	3	3,9	1,7	1,5
1614	250	250,0	250,0	137,5
1670	21	28,6	12,3	10,5
1724	4	5,3	2,3	1,8
1780	1	1,2	0,5	0,45
1785	927	1295,0	559,2	417,15
1793	1	1,3	0,6	0,45
1843	51	70,7	30,5	22,95
1846	74	103,9	44,9	33,3
1853	2250	3141,6	1356,6	1012,462455
1855	308	429,3	185,4	138,6
1856	411	573,6	247,7	184,95
1857	239	332,1	143,4	107,55
1858	230	321,5	138,8	103,6029456
1859	169	236,4	102,1	76,07106011
1860	1564	2183,9	943,1	703,6793586
1861	151	210,9	91,1	67,92893989
1862	227	316,4	136,6	101,9463965
1863	188	262,8	113,5	84,67273475
1864	46	63,8	27,6	20,55883672
1865	133	185,2	80,0	59,85
1867	178,7	249,0	107,5	80,40883672
1869	110	153,6	66,3	49,5
1870	170	237,3	102,5	76,5
1871	455	635,9	274,6	204,7944752
1872	359	495,1	213,8	161,6889447
1873	76	106,3	45,9	34,2
1876	14,0	14,0	13,4	4,35
1877	208	291,2	125,8	93,6
1878	133	185,6	80,1	59,85
1879	148	204,7	88,4	66,6
1880	205	286,3	123,6	92,25
1881	1271	1770,9	764,7	571,884532
1882	607	760,7	328,5	193,6628
1883	78	107,4	46,4	23,96546053
1884	461	644,4	278,3	207,5891447
1885	225	313,8	135,5	101,25
1886	323	451,9	195,1	145,35
1887	234	326,4	141,0	105,3
1888	411	573,9	247,8	184,95
1889	180	250,7	108,3	81
1890	76	106,2	45,9	34,2
1891	136	189,2	81,7	61,2
1892	185	258,9	111,8	83,25
1893	184	256,5	110,8	82,8
1894	133	186,0	80,3	59,85
1895	146	203,5	87,9	65,7
1896	90	126,1	54,4	40,5
1897	111	155,4	67,1	49,95
1898	33	46,6	20,1	14,85

### Literature review

A literature review of published studies about the Order Sirenia and the history of their captures was conducted, following the general approach described by Falcato and Carvalho ^24^. We accessed and utilised major online databases (generic and multidisciplinary) such as JSTOR, SCOPUS, Web of Science, and in different search engines, such as Google Scholar, ProQuest, and B-On. The searches were based on specific keywords. The intention was to generate a global view of the state-of-the-art on the topic and to find and review studies with relevant data. The search was carried out through the Title and Keyword sections, and the parameters used were the same for many established databases and search engines; these are as follows: (manatee OR seacow OR dugong) AND (history OR "early modern") AND (catch* OR Captur* OR harvest* OR fish*); (manatim OR "peixe-boi" OR "vaca-marinha" OR dugongo) AND (historia OR "época moderna") AND (caça OR captur* OR pesc*); (manatí Or “vaca marina” Or dugongo) And (history Or “epoca moderna“) AND (caza OR captur* Or pesc*)ur*). A redundancy process was deployed when publications, screened titles, keywords, and abstracts were perused to ensure that each element was accessed at least twice to minimise oversights or introduced bias, thus maximising the accuracy of identified elements for inclusion and exclusion in the study. Publications written in English, Spanish, Portuguese, and French were included, both indexed peer-reviewed publications and grey literature. We focused on historical data and, consequently, publications of specific thematic criteria such as strictly ecological, behavioural, physiological, and genetic studies were excluded. Also, historical period-based exclusions were implemented to emphasize a focus on the era preceding the Industrial Revolution (c. pre-1860). A cross-check in bibliographic lists of some identified publications was also carried out. From the total of publications included in our reference program on the Order Sirenia worldwide, we identified a total of 292 studies that could be used for qualitative and quantitative analysis and historical contextualization. From these data referring to the Brazil context and for the period beginning with the colonial presence (16 ^th^ century) were used. Raw data was obtained from three established studies – Antunes *et al.* ^25^, Hulsman ^26^, and Domning ^27^.

### Documentary sources analysis

We build on intensive research that has been carried out in recent years and on several historical sources already identified, namely those made available in the online Sea Citation Database ^28^, which includes Portuguese and Spanish documentary sources for the early modern period, ranging from the 16th to the 19th century, and comprises reports, logbooks, descriptions, treaties, bestiaries, natural and general histories, letters, and tax documents. A brief review of Dutch sources was also carried out, since the Dutch occupied territories in north-eastern Brazil between 1630 and 1654 ^29^ and traded manatee products in the South Atlantic ^30^. In total, around 100 historical documentary sources of different typologies were consulted, from which sixteen historical sources were analysed which included letters and descriptions of nature and economic and scientific reports ^1, 26, 31– 43^. Several additional documents from the digital library Projeto RESGATE ^3^ were also analysed and included, namely from the collections ‘Rio Negro’, ‘Pará’ and ‘Geral Brasil’. Sources refer to extractions in the Amazon basin, from its mouth and adjacent waters to the hinterland, leading to our information that refers to the Amazonian manatee ( *T. inunguis*) even though extractions of West Indian manatees ( *T. manatus*) (n = 4) were also recorded in coastal areas of Brazil and Suriname.

Historical documentation can provide information on the harvesting of living marine resources, even for periods when there was no systematic recording of extractions or statistical reports. In many cases, the information is of a qualitative character, which allows us to infer cultural contexts in which the extractions took place, the techniques used, and the people involved, as well as to understand the importance of the animals and the use of their products. In other cases, it is possible to extract quantitative data on the number of animals hunted, even if this information is sometimes scattered. Data are varied in terms of their levels of description and of units used to quantify amounts and products. Meat (dry, salted, or fresh) was generally given in kilograms or equivalent; fat and butter (the so-called ‘mixira’) were packaged in barrels, jars, and cans (all equivalent to each other in terms of content). On occasion, hides were also included, since they were a product to be exported. When available, data with information on the geographic location of captures was considered.

### Mining data and quantification of extractions

To convert the given amounts of manatee products into the numbers of hunted individuals, we followed the methodology used by Domning ^27^ and cross-checked it with other studies, e.g. Whitehead ^44^ and the historical sources available, particularly with Ferreira ^34^. An 'arroba' (an old Portuguese measure of weight) of meat is equivalent to 15 kg; based on the references mentioned above, one jar (‘pote’) corresponds to c. 20–30 kg, and we use the medium value of 25 kg; one can (‘lata’) is equivalent to 22 kg. We used a conversion rate of 76 kg–176 kg of transformed product corresponding to one hunted manatee. Ferreira ^34^ states that for the years 1785–1786 a total of 1500 manatees were hunted and provides the number of products obtained from them. Domning ^27^ states minimum and maximum values for the mass (in kg) corresponding to one animal, which we also use to indicate a confidence interval (or error). We use a conversion factor of 20,34 kg as the average hide weight of commercially hunted manatees, according to Antunes *et al.* ^25^. Based on these conversion rates, we obtained minimum and maximum values for extracted manatees for the region and for the period in question. We obtained a total of 131 data entries in our database ^4^ that were classified under a traffic light code system for the level of certainty of the data obtained. The red entries corresponding to unreliable or unverifiable quantitative information were excluded and only the orange and green data were included, resulting in 52 separate annual records during the period between 1533 and 1898.

### Identification of geographic locations

From the studies and historical sources reviewed, it was possible to determine approximate geographical locations either of the places of capture or the places of trading. When names of villages or broad areas are given, mapping these places offers a spatial visualisation of the extraction practice that can also be combined with other fishing practices or geopolitical settlement information for colonial Brazil. In the 16 ^th^ century Portuguese and Castilians invested in the settlement of the Amazon, first building mission villages, working as indoctrination centers for the natives, under Capuchin, Mercedarian Missionary Sisters, Franciscan, Carmelite, and Jesuit religious orders. In many cases, these mission villages, located precisely in the same places as indigenous societies, settled at the mouths of the Amazon tributaries, where indigenous fishing grounds were located ^45^. Subsequently, these locations were considered strategic for settlement under the Portuguese crown, leading to the construction of forts and villages under the Portuguese colonial administration ^15, 46, 47^. For strategic reasons, the most important Portuguese settlements were located at the confluences of the watercourses, which was also where many of the royal fishing grounds were established ^47^. In fact, identifying possible locations of the first Portuguese settlements and fortifications could indicate possible capture areas, as well as indicate how colonists migrated inland, gaining ground in the Amazon territory ^46, 48^. The need to provide protein, fat and fuel for people who were part of these newly formed colonial communities - crown officials, army, officers engaged in public and civil works, religious missions, members of the expeditions and those determining territorial boundaries - justified the establishment of royal fishing grounds, most on prior indigenous fishing grounds ^17, 19^. With settlements, people, and resources under Portuguese control, former indigenous fishermen worked for the Portuguese crown and under the auspices of royal officials ^15, 49^. Although the distribution of the fishing grounds managed by the Portuguese crown, the so-called royal fishing grounds of the Amazon, are not accurately located, understanding of the capture areas enables insights into the distribution of the capture zones. To reconstitute this geographical distribution, information related to the location of forts and settlements was compiled, considering the chronology of edification. These location data for the royal fishing grounds of the colonial period were gathered by recognising that many had been appropriate indigenous fishing grounds prior to the arrival of the Portuguese, and some of them are still in use today ^17, 19, 50, 51^. Location-extraction datasets were considered and included in the georeferencing elements of the database. Extraction records for undefined areas, such as the Amazon province, were mapped within an extended area corresponding to the region.

## Results

Here we focus on preindustrial captures and the long-term effect of continued extractions of manatees by colonial Europeans in tropical regions, up to end of the 19 ^th^ century.

(…) *at eight o'clock we arrived at the harbour of the said town* [village and fortress of Obidos, Brazil], *which is thirty-two leagues away from the upper mouth of the Surubiú River, from where we set sail on the 21st: soon afterwards, H.E. disembarked, having the pleasure of seeing on the beach three manatees, which had been caught that morning* (...) *from there he went to examine the pottery and warehouses of the same village* [of Santarém] *, then to see its beaches, and at the house of the administrator of the royal fishing grounds to observe a live manatee that was kept there, as well as two dead ones that the fishermen from the town had brought that night* (...) ^52^. ^5^

### Extractions and biomass removal

**Figure 2.**
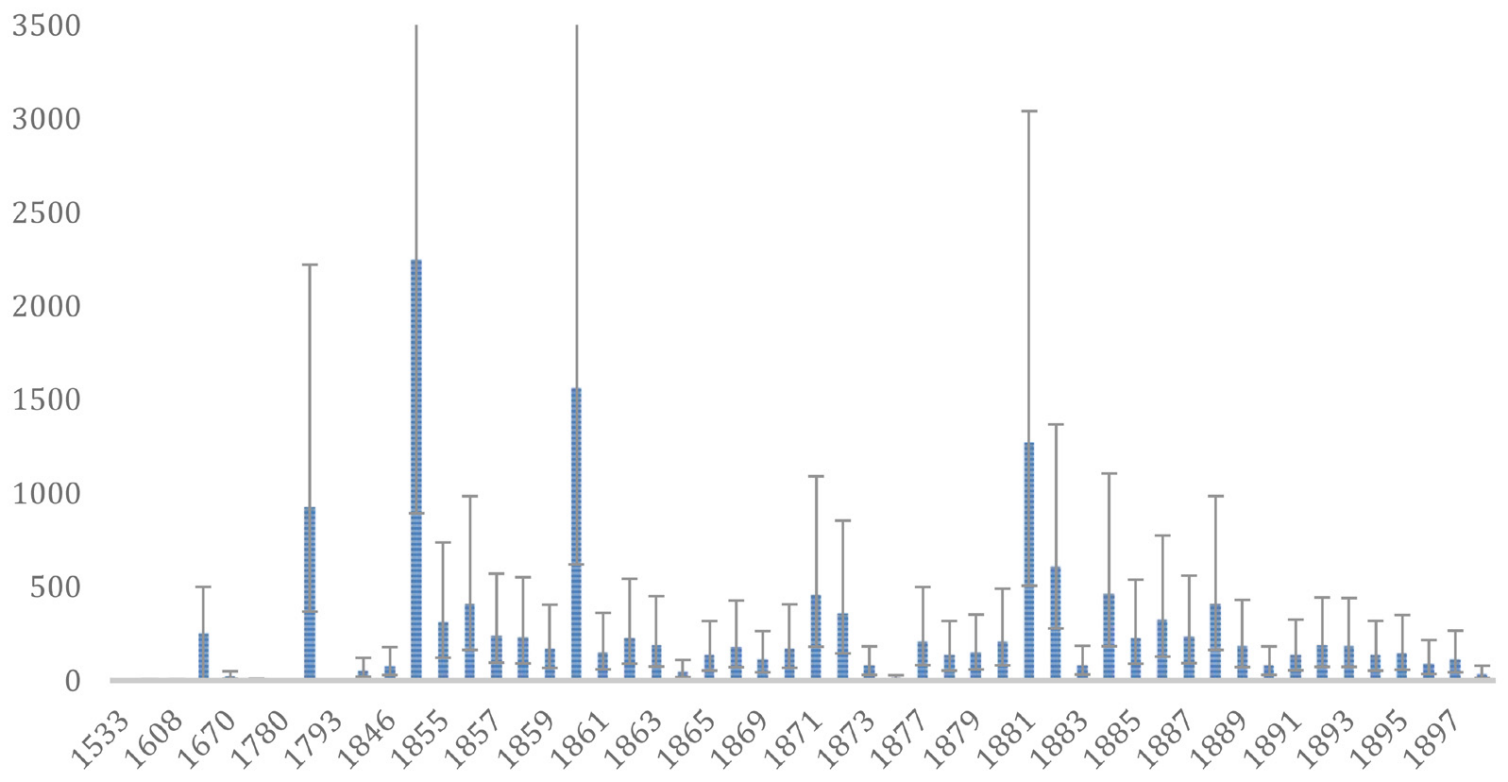
Documented years (n=52) of manatee extractions ( *Trichechus* spp.): 1533 to 1898. Data are for the Amazon basin and adjacent waters, except for 1533 (southern coast of Brazil), 1608 and 1670 (Suriname) (see supplementary material online). The average number of manatees extracted are indicated by the blue bars, showing the variation of maximum and minimum estimates.

**Figure 3.**
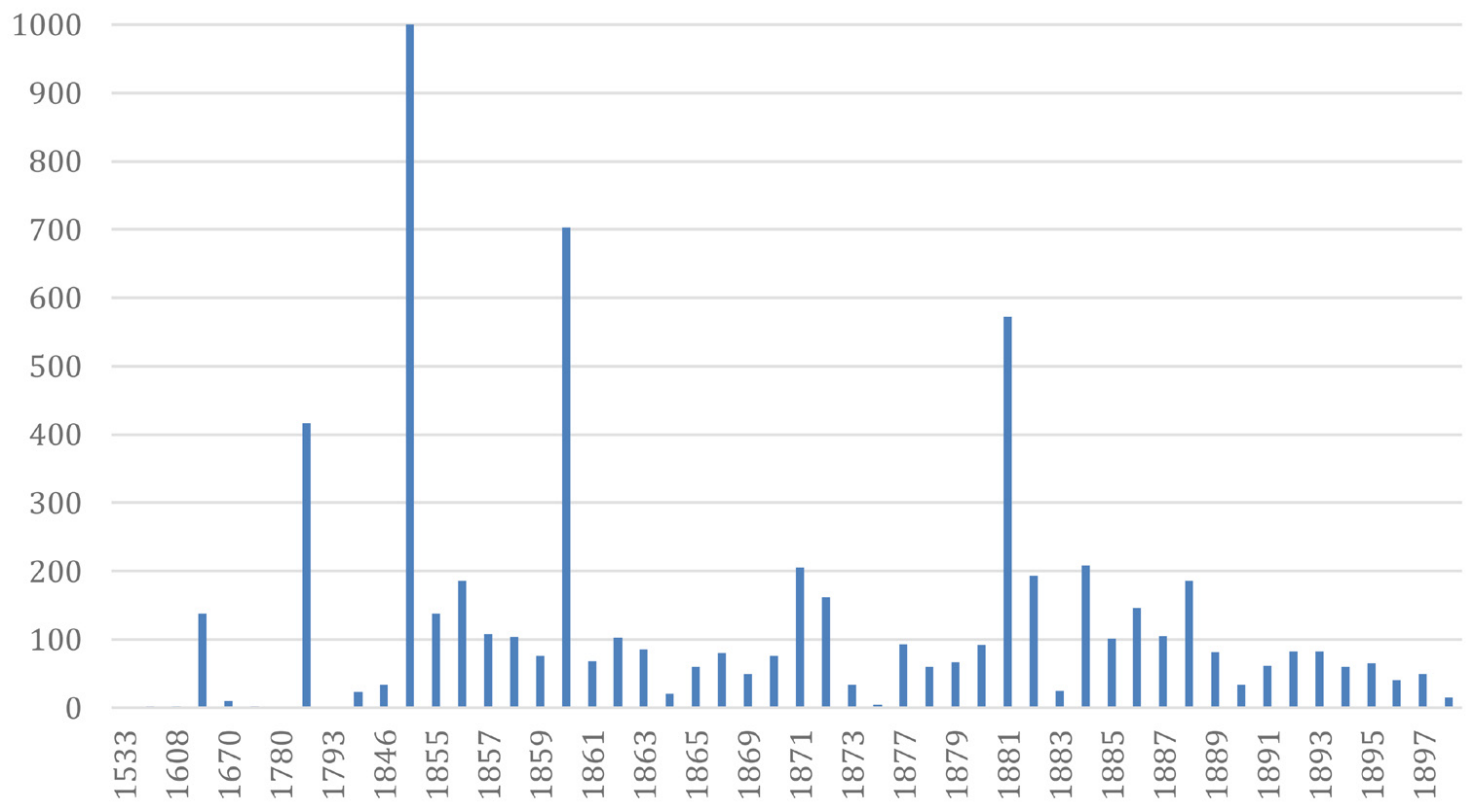
Removal of manatee biomass ( *Trichechus* spp.) for the period between 1533 and 1898, in tons. Data are for the Amazon basin and adjacent waters, except for 1533 (southern coast of Brazil), 1608 and 1670 (Suriname) (see supplementary material).

Our quantitative estimate resulted in a total of 14.030 (min. 8.301 – max. 19.220) manatees extracted in a total of 52 years during the 16 ^th ^(n=1), 17 ^th^ (n = 4), 18 ^th^ (n = 4), and 19 ^th^ (n=43) centuries; 'n' is the number of years for which quantitative data is available. These values represent the period between 1533 and 1793, and almost continuously from 1843 to 1898 ( Figure 2 and Table 1). A total of 12.818 hunted manatees are indicated for the period between 1843 and 1898, comprising 91% of the captures. The oscillations observed over the centuries can be assumed as a global trend that is consistent with Domning ^27^ and Antunes *et al.* ^25^, justified by the inconsistency of historical data, and that is also the case for other species of marine mammals, such as cetaceans ^53^. In addition, we estimated the removal of manatee biomass ( *Trichechus* spp.) for the study period (1533–1898) as a first attempt to develop repeatable and reliable methods to estimate live weight biomass from historical data. Based on our estimates of individuals captured and derived weight for the species at 550 kg for *T. manatus* ^54^ and at 450 kg for *T. inunguis* ^54– 57^, we obtained a total of 6.248,9 tons ( Figure 3).

### Historical trends in abundance and geographic distribution

Our comparison of multi centuries extractions shows a trend of increasing captures from the 17 ^th^ to the 20 ^th^ centuries; for the 16 ^th^ century one single source was found.

**Figure 4.**
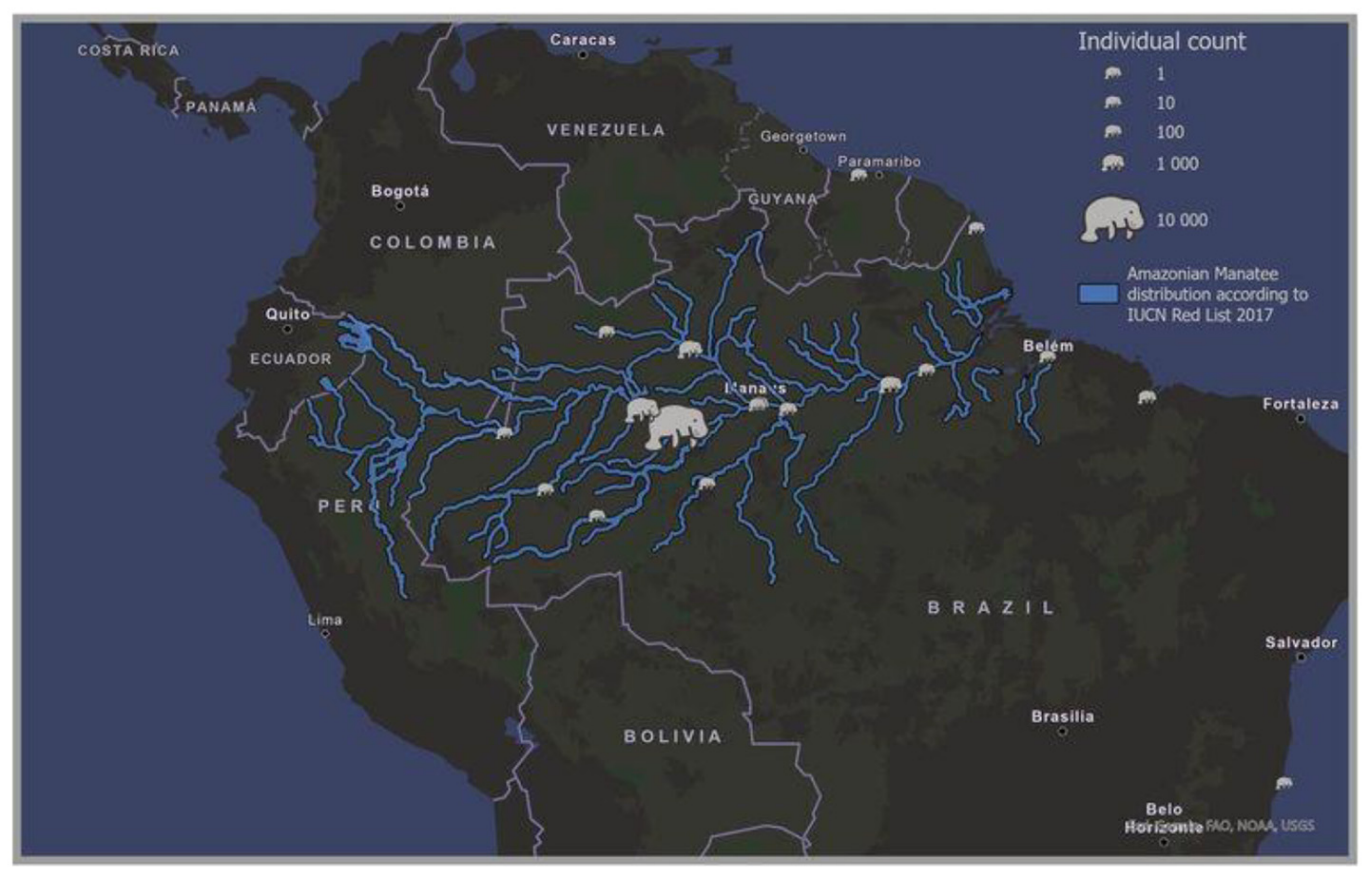
The extent of Amazonian manatee exploitation over c. 400 years mapped across the current species geographic distribution. The blue lines indicate the current geographic distribution of the Amazonian manatee ( *Trichechus inunguis*) (The IUCN Red List of Threatened Species). https://www.iucnredlist.org), and symbols show locations of extractions from 1533 to 1898 ( *Trichechus sp.*). Mapped in ArcGIS [GIS software].

To better understand the intensity and geographical areas where manatees were taken, information was provided on the European colonisation of the Amazon region. The geographical extent of the exploited manatees ranged from the Brazilian coast (from Fort São Joaquim do Rio Branco in the north and the Royal Fort of Príncipe da Beira in the south), but mainly around the Amazon basin and delta and the various tributaries of the Amazon upstream to Tabatinga near the Peruvian border, a conflict zone with Castilian settlements. The two species occur in this geographical area, with a possible overlap in the nearshore waters of the Amazon estuary. In mapping extractions of manatee locations for the total analysis period, the middle Amazon was found to be the primary region where manatee products were traded ( Figure 4). This region is where the bulk of captures occurred, and this is a good indicator that the dominant species of manatee that was hunted is *T. inunguis*.

**Figure 5.**
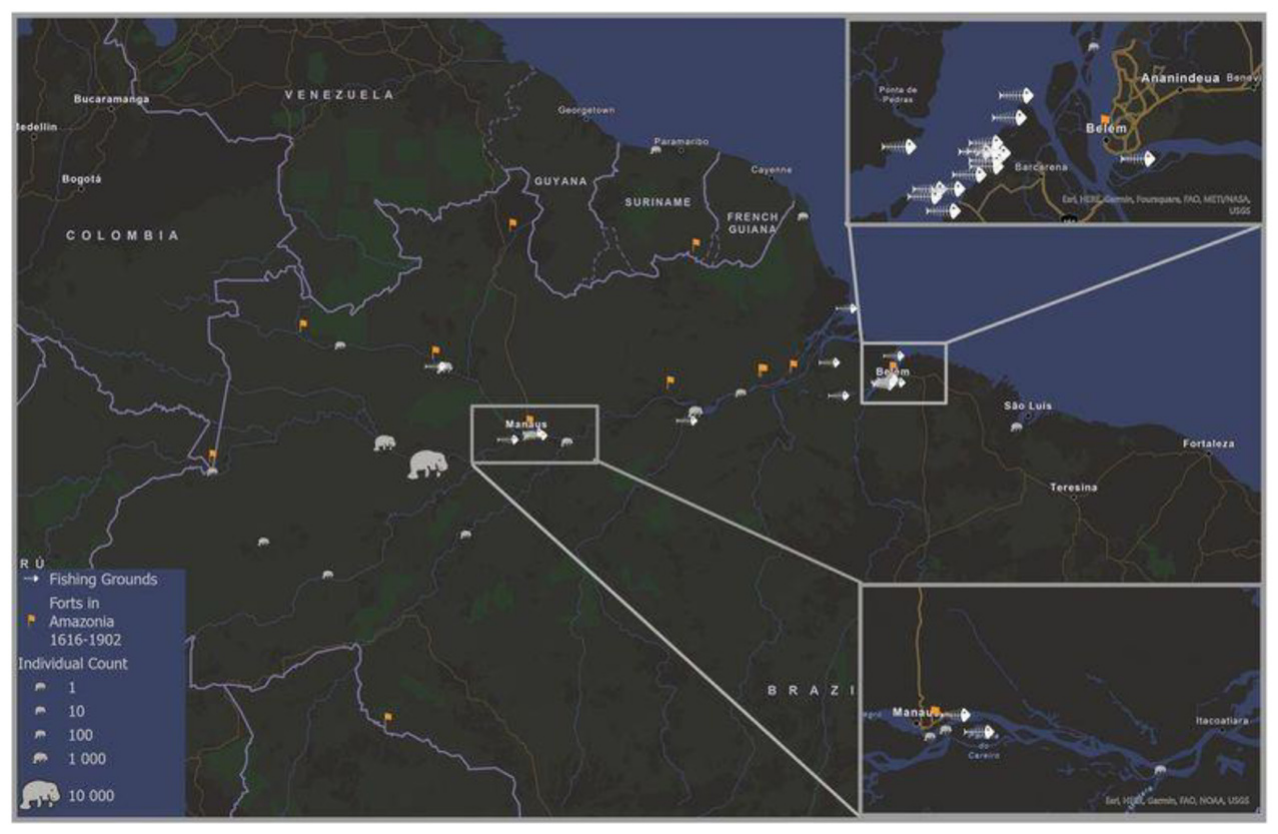
Locations of manatee extractions plotted over historical fishing grounds (´Pesqueiros’) and forts in the region; Zoom in for the Manaus and Belém do Pará regions are shown. Mapped in ArcGIS [GIS software].

The conquest movement also shows locations where natural resources, including manatees, were needed the most. Through our mapping of the settlements, we recognise that military control posts penetrated further inland, and the occupation of the territory became more intense throughout the 18 ^th^ century. As previously identified, the so-called royal fishing grounds, under Portuguese domain, were in many cases former indigenous fishing grounds. Many of the actual fishing grounds continued to be used, and even today names such as *Pesqueiro dos Manatins* (manatee fishing grounds) and *Pesqueiros das Tartarugas* (turtle fishing grounds) are still used. After the Portuguese administration took over, the fishing grounds began to provide these communities with protein and enabled them to generate surpluses for export through increasingly intensive fishing. The approximate geographical allocation of manatee catching or export areas along the Amazon corresponds in many cases to important military zones such as Belém do Pará and Manaus ( Figure 5). Observation of the geographical location of the fishing grounds in their vicinity indicates that there is a correlation between the three elements of fortifications, fishing grounds, and manatee catches. From the geographical distribution, we can see that captures follow the process of occupying the hinterland alongside the establishment of Portuguese military positions.

## Discussion

### Pre-industrialization extractions of Amazonian manatees

Continued exploitation of aquatic animals led to continued use and demand for key species of interest, structured around the mindset and ideology of European property and rights of exploitation, even if the system was built upon local practices and knowledge about the aquatic resource and their ecosystems. Manatees were historically used locally as food, medicine, and mythical-religious objects by the native peoples of the Amazon region. Colonisation across the South Atlantic led to the development of regional and transoceanic networks built to feed European settlers and their multiple demands within colonial systems and dynamics. With the opening of overseas territories to European societies because of early modern expansions and the so-called early globalisation, a new set of cultural, economic and ecological perspectives emerged.

A sense of novelty, high biodiversity, and infinite abundance permeate historical sources that refer to aquatic animals of the Americas. In a letter of 1560, two manatees captured off the coast of Brazil are reported ^1^. By 1592, Portuguese ships traveling the Atlantic would stop in Brazil to take several barrels of salted manatee meat that were hunted in the region ^58^. In the middle of the 17 ^th^ century, the Dutch, who at the time occupied various regions of northeast Brazil, traded with indigenous groups, namely the Nheengaíbas ^43^. Even if not allowing us to quantify the extent of the catches, this type of qualitative data allows us to understand their use and importance in tropical and subtropical colonial contexts. Manatees became food items within colonial societies in the Americas ^41^. Attempts were made to send barrels of manatee meat to Lisbon, using the salting method for other types of meat ^59, 60^. Manatee hunting continued after Brazil's independence in 1822 and intensified in the postcolonial period. Two accelerated extraction epistemes are identified. From the 19 ^th^ century onwards, all parts of the animal were deemed useful, meat for food, fat for oil production, bones for making various objects of daily use, such as hooks and necklaces, as well as the leather used, for example, to make whips ^61, 62^. In the second half of that century, there were newspaper advertisements for manatee skin with curative effects for hernias, ruptures, and asthma ^63^. In the 20 ^th^ century a pronounced increase in the capture of manatees emerged ^25^ along with many other aquatic species, with dramatic impacts on the maintenance of populations. It was an accelerated period of ocean extraction and exhaustion ^21, 64, 65^, based not only on demand but also on technological innovations (namely motorization of boats) built on previous continued exploits.

Spatial control by European nations over local indigenous ones also reflects the value of the aquatic animals as resources for different peoples. Historical geographic information shows the different regions of exploitation and extraction in the Amazon following the increasing need for local resources to feed the local society ^46, 62, 66– 68^. In fact, the Portuguese fortifications, hinterland movements, and fishing grounds established offer an insight into the evolution of the colonial presence in the Amazon. The increased settlement and colonisation are paralleled by the levels of exploitation of manatees in terms of numbers and locations along the Amazon River. The importance of this aquatic resource to colonial and postcolonial societies is highlighted by the close relationship between population growth, impacts, and exploited manatee numbers.

Colonisation and appropriation of peoples, lands, and resources became common practice, disrupting not only local societies and ways of living, but also natural populations and ecosystems. Marine and freshwater resources from subtropical and tropical ecosystems are among those that caught the interest of new local colonial societies and the global and imperial interests of European nations. These practices of extraction had long-term ecological and sociocultural impacts – regional extirpations and symbolic disruptions.

### Discussing historical trends of exploitation of manatees

The global overview of the quantitative data obtained in this work offers a broad view of the global temporal trend of increasing exploitation with peak moments. Changes in the numbers of captured manatees over the time may indicate: 1) natural fluctuations in the local populations that directly connect to resource availability; 2) environmental constraints that influence local habitats and extraction practices; 3) overexploitation, local extirpations, and the constraining of areas of occurrence; 4) socioeconomic need to produce surplus due to demographic changes and the necessity to respond to local, regional, and long-distance demand. In combination, they indicate processes of colonial and ecological imperialism and oceanic teleconnections ^64, 65, 69^. We could further discuss hypothetical estimates of the ‘true’ scale of hunting based on an extrapolation of the data obtained for the data-sourced 52 years for the wider period of c. 400 years. That is, we can account for estimates of at least eight times higher the number obtained, meaning values of manatees’ extractions of c. 110.000 specimens extracted. But more relevant than exact number of past extractions – that were significative and impactful –, is to address the fact Amazonian manatee population decreased significantly over a period of four centuries. Even if underestimated these data are relevant for assessing population trends and status. To date, genomic and survey data suggest that the overall population trend for this species is one of decline, however the full temporal and spatial extent of this decline is an ongoing endeavour. Our study highlights the importance of mining historical sources and the value of historical data (both descriptive and numeric) to quantify the removal of aquatic biomass in the past. In future studies of the historical aquatic ecology of the Amazon, the general extraction of biomass from the environment should be considered, the correlation between the extractions with environmental and climatic data, namely changes in the course and amplitude of river flows, and times of flood or drought.

In terms of human practices, hunting techniques and uses of these animals do not appear to have changed in Brazil over centuries of their exploitation ^3, 70^. Currently, capture is largely motivated by appreciation for the taste of manatee meat and its economic value on the market, and although communities do not rely on this animal protein for their subsistence, the importance of the tradition of manatee consumption for the local population should not be underestimated ^3^. This is also true for other endangered or protected aquatic mammals in this and other regions ^71, 72^. The Amazon manatee - in fact, the three species of the genus *Trichechus* - is listed as ‘vulnerable’ by the IUCN based on a predicted population decline of at least 30% within the next three generations (generation duration 25 years) ^73^. The species is included in Appendix I of the CITES and several conservation efforts have also been made both regionally and nationally. ^6^

As a process of ecological imperialism and globalisation ^74– 76^, intensive extractive practices transformed manatees into commodities in colonial territories that contribute to changes in their distribution and abundance ^77^. Colonial hunting disrupted indigenous systems of capture and use of resources and of symbolic meanings attributed to manatees as part of local eco-cultural realities ^2^, as much as it has disrupted ecological niches. Today, manatees are part of local and global histories and are symbols of endangerment and nature degradation, but they are also examples of recovery, of local empathy between peoples and animals, and part of traditions that go beyond a westernised view of nature conservation. By encompassing information and contexts of the past, the possibility of ethnohistorical and multi-specific relationships, and multiplicity of ecocultural systems in which manatees and humans cohabit, effective mechanisms of dealing with potential extirpations and the much-desired maintenance of healthy populations of aquatic animals may be achieved.

## Ethics and consent

Ethical approval and consent were not required.

## Data Availability

All historical sources and data collected in this study are available in online, open-access format that summarises the data sets and provides statistical information (DOI: https://doi.org/10.5281/zenodo.15739198). We have made all data publicly available following the Darwin Core scheme, ensuring the inclusion of relevant data fields. It is openly shared, distributed or used under Creative Commons licensing provisions CC- BY 4.0.
