# Supplementary material for: Analyses of historical documents reveal past trends of exploitation of manatees ( Trichechus inunguis) in the Amazon Basin (16th-19th centuries)
Source: Open Res Eur. 2025 Aug 18;5:245. [Version 1] doi: 10.12688/openreseurope.20811.1 (PMC12592855; doi:10.12688/openreseurope.20811.1)
Supplement: Peer review history

# Reviewer report: Reviewer response for version 1

**DOI:** 10.21956/openreseurope.22516.r63132

**Published:** 2025-11-06

**Authors:** Felipe Vander Velden

**Affiliations:** 1 Federal University of São Carlos, São Carlos, Brazil

The article under reviewed is quite interesting and highly informative, as it presents a combined reading of quantitative and qualitative data that allows access to aspects of the history of relationships between human communities (pre- and post-Columbian) and manatees in the Amazon. Thus, I agree that this study indeed sheds light on the “importance of mining historical sources and the value of historical data (both descriptive and numeric) to quantify the removal of aquatic biomass in the past” and, in doing so, helps us better understand the current situation of these species regarding their conservation status.

 I have only a few comments/suggestions to make, which I believe are meant to improve the text. First, I think it would be useful for readers if the article included information about manatee ecology and behavioral characteristics, as such data could help clarify methods, locations, and capture preferences. Second, I did not fully understand the idea that manatees became “symbolic elements” in the establishment of human interactions with the natural world. What symbolic elements are these? And which “local myths and legends” about manatees exist in the Amazon, since this information is not referenced? Furthermore, I find it somewhat exaggerated to say that manatees are part of “Brazilian folklore”, given that they are largely unknown in other parts of the country outside the Amazon and the northeastern coast. Third, I would also appreciate references to the claim that manatees have become “examples of recovery, of local empathy between peoples and animals, and part of traditions that go beyond a westernized view of nature conservation,” since this statement is also not supported by citations.

 Finally, I think the article deserves a careful review of the English. Nevertheless, it is a substantial, original, informative, and thorough piece of work, and should therefore be indexed.

If any, are all the source data and materials underlying the results available?

Yes

Is the work original in terms of material and argument?

Yes

Is the work clearly and cogently presented?

Yes

Is the argument persuasive and supported by evidence?

Yes

Does the research article contribute to the cultural, historical, social understanding of the field?

Yes

Does it sufficiently engage with relevant methodologies and secondary literature on the topic?

Yes

Reviewer Expertise:

Amazonian Ethnology; Anthropology of Human-Animal Relations

I confirm that I have read this submission and believe that I have an appropriate level of expertise to confirm that it is of an acceptable scientific standard.

# Reviewer report: Reviewer response for version 1

**DOI:** 10.21956/openreseurope.22516.r58693

**Published:** 2025-09-16

**Authors:** Yann Hénaut

**Affiliations:** 1 El Colegio de la Frontera Sur (ECOSUR), Quintana Roo, Mexico

This work presents highly original data on the history of Amazonian manatee exploitation. It provides new and very interesting information on this subject, particularly with regard to periods prior to the present day (before the nineteenth century) and how this historical exploitation has impacted on the current conservation status of the species.

 Overall, the article seems well written, but as I am not a native English speaker, I cannot judge the details of the language.

 The methodology also seems comprehensive to me, although I am not a specialist in this type of study.

 I believe this work deserves to be indexed and will shed new light on our history in relation to the conservation of manatees.

If any, are all the source data and materials underlying the results available?

Yes

Is the work original in terms of material and argument?

Yes

Is the work clearly and cogently presented?

Yes

Is the argument persuasive and supported by evidence?

Yes

Does the research article contribute to the cultural, historical, social understanding of the field?

Yes

Does it sufficiently engage with relevant methodologies and secondary literature on the topic?

Yes

Reviewer Expertise:

Animal behavior

I confirm that I have read this submission and believe that I have an appropriate level of expertise to confirm that it is of an acceptable scientific standard.
